# Tamoxifen use and risk of endometrial cancer in breast cancer patients: A systematic review and dose–response meta‐analysis

**DOI:** 10.1002/cnr2.1806

**Published:** 2023-03-14

**Authors:** Matin Ghanavati, Yasaman Khorshidi, Mahdi Shadnoush, Mohammad Esmaeil Akbari, Seyed Hossein Ardehali, Yanin Chavarri‐Guerra, Atieh Akbari, Regina Barragan‐Carrillo, Manoochehr Amin Amlashi, Zeinab Javid, Jamal Rahmani

**Affiliations:** ^1^ Department of Clinical Nutrition & Dietetics, Faculty of Nutrition Science and Food Technology, National Nutrition and Food Technology Research Institute Shahid Beheshti University of Medical Sciences Tehran Iran; ^2^ Cancer Research Center Shahid Beheshti University of Medical Sciences Tehran Iran; ^3^ Department of Anesthesiology and Critical Care, Shohadaye Tajrish Hospital Shahid Beheshti University of Medical Sciences Tehran Iran; ^4^ Department of Hemato‐Oncology Instituto Nacional de Ciencias Médicas y Nutrición Salvador Zubirán Mexico City Mexico; ^5^ Department of Nutrition, School of Public Health Iran University of Medical Sciences Tehran Iran; ^6^ Department of Nutrition, Farhikhtegan hospital, Faculty of Medicine, Tehran Medical Sciences Islamic Azad University Tehran Iran

**Keywords:** breast cancer, dose–response, endometrial cancer, tamoxifen

## Abstract

**Background:**

Worse prognosis of endometrial cancers (EC) in tamoxifen‐treated women compared to non‐tamoxifen‐treated women been proposed. The relationship between tamoxifen treatment of breast cancer (BC) and the risk of EC is controversial and there is no agreement between publication results on this issue (the answer to all comments provided in the page 2 of manuscript). The aim of this study is investigation the association between tamoxifen treatment and the risk of EC in patients with BC.

**Methods and Results:**

We conducted a comprehensive search with related keywords in MEDLINE/PubMed, SCOPUS, and Web of Science databases until April 16, 2022. Random‐effects model (DerSimonian and Laird) was used to pool risk ratios (RRs) with 95% confidence intervals (CIs) of EC. Dose, cumulative dose, and duration‐response analysis were performed in linear and non‐linear states. Twenty‐six studies reported a relation between tamoxifen treatment and risk of EC in patients with BC. Results showed a direct relationship between tamoxifen use and EC (RR: 2.03, 95% CI: 1.68–2.45; I2:76%). By increase the age of participants, the risk of EC was decrease (coef = −.0206), although this was not statistically significant (*p* = .37). Linear dose–response model indicated a direct significant association between dose and duration use of tamoxifen and EC (dose: exe(*b*) = 1.019, *p* = .001; duration: exe(*b*) = 1.014, *p* = .001). Non‐linear dose–response analysis confirmed linear analysis.

**Conclusion:**

This study highlights that tamoxifen use is a significant risk factor related to the incidence of EC in patients with BC.

## 1 BACKGROUND

Hormone‐receptor‐positive breast cancer (BC) is the most frequent biologic subtype of breast malignancy, accounting around 75% of new BC diagnoses.^1^ Tamoxifen, an oral selective estrogen receptor modulator (SERM), is currently one of the mainstays for treating hormone‐receptor‐positive BC in adjuvant, especially in perimenopause women.^2^, ^3^, ^4^ Evidence regarding the benefit of tamoxifen extended adjuvant therapy for longer than 5 years has emerged, especially for high‐risk patients with an overall risk reduction for recurrence, BC related‐ and widespread death.^5^, ^6^, ^7^

Tamoxifen's particular mechanism of action is characterized by its activity as a competitive antagonist of the alfa‐estrogen receptor within breast tissue, nonetheless as an agonist in endometrial tissue with a dose‐ and duration‐dependent effect.^8^ The reflected effects of the beta‐estrogen receptor within the endometrium explain the increased incidence of endometrial pathology including hyperplasia, atypia, or malignancy. The risk for associated uterine malignancy increases after long‐term exposure, mainly in postmenopausal women with preexisting pathologies in the uterus.^9^ Prior evidence regarding endometrial cancer (EC) risk associated with tamoxifen intake in the younger patient population as some data have pointed out an increased risk of endometrial neoplasia associated with the SERM. In contrast, other data have not reproduced these findings.^10^

An investigation of the relationship between tamoxifen use and EC in patients with BC was published by Choi et al.^11^ They examined 256 099 patient‐years in which 140 patients developed EC. BC survivors taking tamoxifen tend to develop EC. Patients with aged 60–59 years, 50–59 years, and 40–49 years showed hazard ratios of 5.03 (95% Cl: 2.18–11.61), 4.34 (95% Cl, 2.12–8.89), and 2.12 (95% Cl: 1.06–4.21), respectively.^11^ Another study by Chu et al. examined the risk of EC in 14 588 nonusers and 19 302 users of tamoxifen over 14 years.^12^ Users had a higher risk of developing EC than nonusers HR 3.90 (95% CI: 2.37–6.42) (1.7% vs. 0.3% incidence). However, Chiofalo et al. conducted a study in 2020 to examine the risk of EC among BC survivors taking aromatase inhibitors, tamoxifen, or receiving no treatment at all, and the results indicate that tamoxifen users did not seem to have a higher risk of EC compared to aromatase inhibitor users or those who did not receive treatment.^13^ Different studies investigating the relationship between tamoxifen and EC produce contradictory results.^11^, ^12^, ^13^, ^14^

The current work represents the most updated data regarding the association between tamoxifen exposure and EC. We emphasize age of participants, dose of tamoxifen, cumulative dose of tamoxifen, and duration of tamoxifen use.

## 2 METHODS

### 2.1 Data search strategy

In this systematic review and meta‐analysis, the preferred reporting items for systematic reviews and meta‐analyses (PRISMA) were followed.^15^ We conducted a search in databases (PubMed, WOS, and Scopus) on April 16, 2022, with English language and without time restriction. The electronic database search strategy and related words are provided in Table S1. The reference list of the original studies included was reviewed to identify additional studies.

### 2.2 Inclusion and exclusion criteria

Studies that met this study's inclusion and exclusion criteria remain in the final analysis. The inclusion criteria were: (1) cohort, case‐cohort, case–control studies, or randomized controlled trial (RCT) designs; (2) studies that reported a significant or non‐significant (null findings) relation between tamoxifen use and EC in patients with BC; (3) EC occurred after BC; (4) studies with appropriate estimates such as hazard ratio (HR), risk ratio (RR), or odds ratio (OR) and corresponding 95% confidence intervals (CI) or at least those that provided data to calculating the estimates. Other type studies excluded. Furthermore, studies with inappropriate or insufficient data, studies without control groups, and studies without full text were excluded. The reports with the highest quality or the most prolonged follow‐up period were used in multiple reports data.

### 2.3 Data extraction and quality assessment

Two independents' authors reviewed titles/abstracts and full texts. Data extraction was performed using a prepared form. Following data were extracted from included studies: authors, publication year, country, study design, follow‐up time, number of study population, number of cases, age, race, ethnicity, duration and dose of tamoxifen, cumulative dose of tamoxifen, details of EC, and summary estimates and 95% CIs of EC risk. If studies provided several models to estimate risk ratio, fully adjusted models were used in the analysis. Newcastle‐Ottawa Quality Assessment Scale (NOS) was used to assess the quality of the observational studies, and the Cochrane Collaboration's tool was used for risk of bias assessment in randomized trials.^16^, ^17^

### 2.4 Statistical analysis

STATA 14.0 statistical software (Stata Corporation, College Station, Texas, United States) was used to perform all statistical analyses. A *p* value <.05 was considered significant. Random model (DerSimonian and Laird) run to pool the results.^18^ Non‐user category of tamoxifen was considered as the reference. The heterogeneity among studies was estimated using the Cochran Q test (P heterogeneity) and I2 statistic.

The I2 metric was used to estimate heterogeneity among studies (50% considered as cut‐point of heterogeneity). Restricted cubic splines run to investigate the potential curvilinear relation among tamoxifen use and RR of EC among patients with BC (three knots at percentiles of 10%, 50%, and 90% of the distribution).^19^ Sub‐grouped analysis and meta‐regression were used to find the source of heterogeneity. To test the curve‐linearity of the dose–response meta‐analysis, *p* values were calculated using the null hypothesis that the coefficient of the second spline was zero. To determine whether a single study would affect pooled results, a sensitivity analysis was conducted. An investigation of publication bias among studies was conducted using the funnel plot, Begg's and Egger's tests. Trim‐and‐fill analyses were used to examine the effect of publication bias. Both‐armed zero‐event studies excluded from analysis, but Single‐arm zero‐event studies evaluated with addition of 1 either to all cells of the problematic tables or to all cells of all studies.

## 3 RESULTS

### 3.1 Search results

**FIGURE 1:**
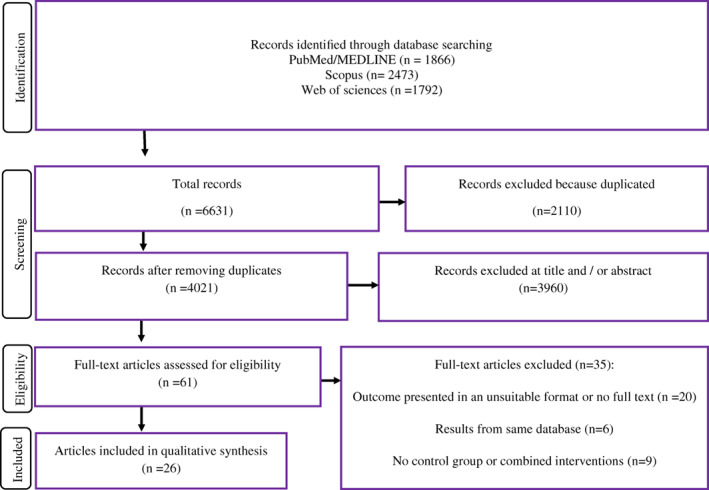
Flowchart of study selection.

Figure 1 provides the flow diagram of included studies. The systematic search provided 6631 studies and 2110 of them were duplicates. According to the inclusion criteria, in title/abstract and full text screening, 3960 and 35 studies were excluded, respectively. Finally, 26 studies were eligible for inclusion in meta‐analysis.^11^, ^12^, ^13^, ^14^, ^20^, ^21^, ^22^, ^23^, ^24^, ^25^, ^26^, ^27^, ^28^, ^29^, ^30^, ^31^, ^32^, ^33^, ^34^, ^35^, ^36^, ^37^, ^38^, ^39^, ^40^, ^41^

### 3.2 Study characteristics and quality assessment

Table 1 shows the main characteristics of the included studies. A total 26 studies with 238 394 individuals and 3280 cases were investigated. Included studies were published between 1988 and 2021. Mean age of participants was 58.4 years. Five studies were performed in the United States,^24^, ^25^, ^30^, ^36^, ^40^ one in Canada and United States,^22^ two in Italy,^13^, ^14^ three in Japan,^28^, ^33^, ^38^ two in France,^27^, ^29^ two in Sweden,^20^, ^26^ two in Finland,^23^, ^35^ two in the United Kingdom,^37^, ^41^ and others in South Korea, Taiwan, United Kingdom, Netherlands, Slovenia, Austria, Israel, and Denmark.^11^, ^12^, ^21^, ^31^, ^32^, ^34^, ^39^ Three studies had RCTs design,^21^, ^22^, ^26^ eight studies had case–control design,^20^, ^24^, ^27^, ^29^, ^30^, ^32^, ^35^, ^37^ and 15 studies were cohorts.^11^, ^12^, ^13^, ^14^, ^23^, ^25^, ^28^, ^31^, ^33^, ^34^, ^36^, ^38^, ^39^, ^40^, ^41^ More details about number of tamoxifen user in each group provided in the Table S2.

**TABLE 1:** Characteristics of trials and participants.

First author	Year	Country	Study design	Number of populations (*n*)	Number of case (*n*)	Age (Year)	Study duration
Portela, S.	2021	United Kingdom	Cohort	94	45	65	2008–2018
Choi, S.	2021	South Korea	Cohort	60 545	140	51	2009–2018
Chu, S. C.	2020	Taiwan	Cohort	33 890	116	49	1999–2012
Chiofalo, B.	2020	Italy	Cohort	1067	28	54	2007–2016
Guerrieri‐Gonzaga, A.	2016	Italy	Cohort	883	5	52	1996–2008
Chlebowski, R. T.	2015	United States	Cohort	7974	107	68	1991–2010
Lavie, O.	2008	Israel	Cohort	1496	14	58	1987–2004
Yamazawa, K.	2006	Japan	Cohort	674	6	‐	1989–1998
Swerdlow, A. J.	2005	United Kingdom	Case–control	1880	813	61	1976–1996
Curtis, R. E.	2004	United States	Cohort	106 641	888	66	1980–2000
Pukkala, E.	2002	Finland	Case–control	497	140	‐	1980–1995
Vrscaj, M. U.	2001	Slovenia	Cohort	630	13	65	1987–1994
Matsuyama	2000	Japan	Cohort	6026	12	51	1982–1996
Bergman, L.	2000	Netherlands	Case–control	1159	299	65	1972–1997
Peters‐Engl, C.	1999	Austria	Cohort	4109	25	‐	1975–1995
Bernstein, L.	1999	United States	Case–control	995	324	65	1978–1992
Mignotte, H.	1998	France	Case–control	622	135	58	1976–1994
Katase, K.	1998	Japan	Cohort	825	13	50	1980–1990
Sasco, A. J.	1996	France	Case–control	220	43	56	1982–1992
Rutqvist, L. E.	1995	Sweden	RCT	2729	27	‐	1976–1990
Robinson, D. C.	1995	United States	Cohort	586	8	53	1978–1989
Cook, L. S.	1995	United States	Case–control	98	34	63	1978–1990
Lahti, E.	1994	Finland	Cohort	105	5	63	‐
Fisher, B.	1994	Canada and the United States	RCT	2843	17	‐	1982–1988
Andersson, M.	1992	Denmark	RCT	1710	9	‐	1977–1982
Hardell, L.	1988	Sweden	Case–control	96	14	‐	1971–1976

*Note*: Year denotes the year of publish study.

Abbreviation: RCT: randomized controlled trial.

Quality assessment of the included studies is provided in the Table S3. The quality score of cohort studies were from 5 to 9, and for case–control studies from 7 to 9, and RCTs from 4 to 5. The main issue about quality of cohort studies was “control for important factor or additional factor” item and this issue was “non‐response rate” for case–control studies, and “blinding of outcome assessor” in RCTs.

### 3.3 Main results

**FIGURE 2:**
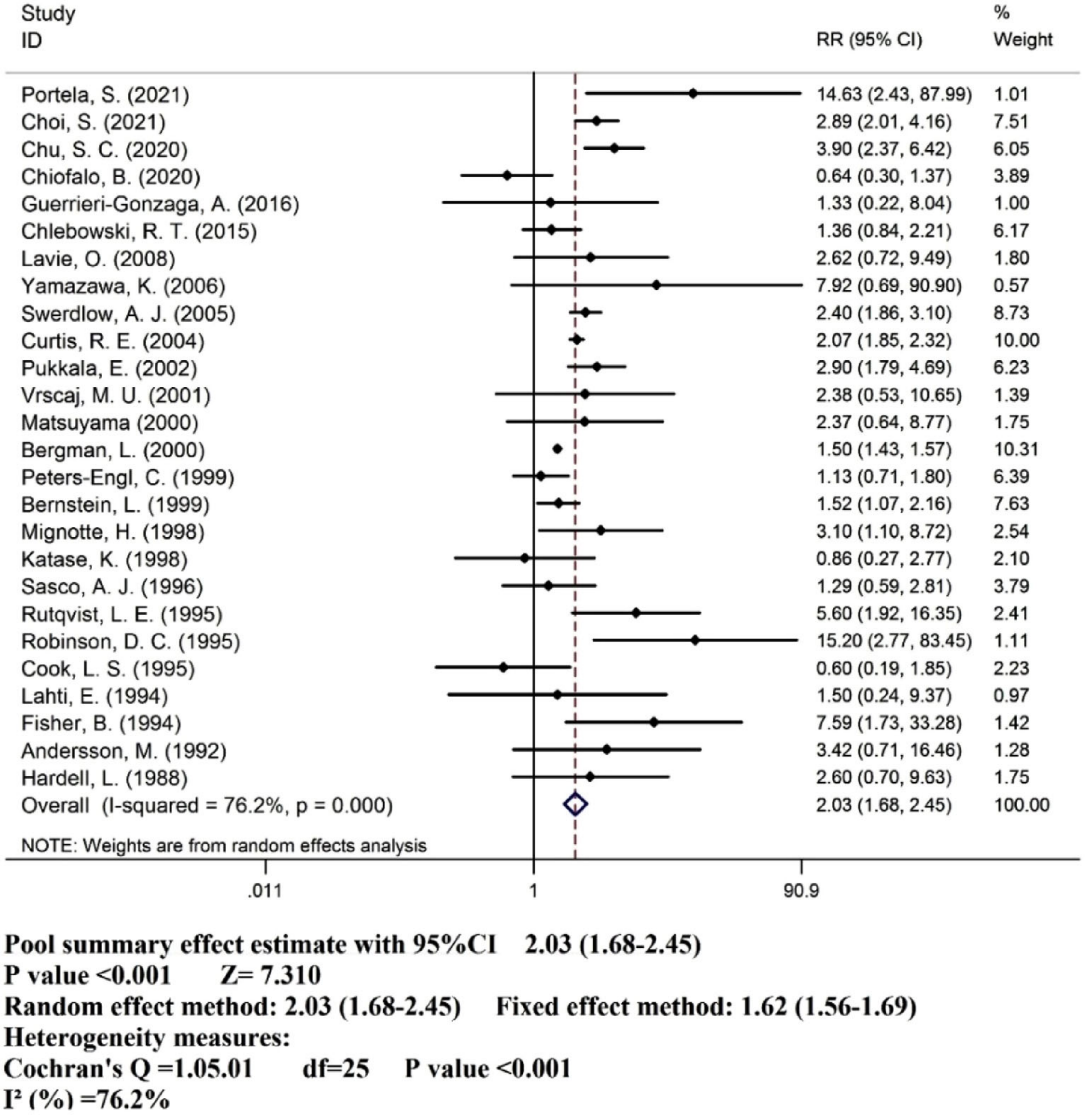
Risk ratios for endometrial cancer after tamoxifen treatment of breast cancer.

Twenty‐six studies^11^, ^12^, ^13^, ^14^, ^20^, ^21^, ^22^, ^23^, ^24^, ^25^, ^26^, ^27^, ^28^, ^29^, ^30^, ^31^, ^32^, ^33^, ^34^, ^35^, ^36^, ^37^, ^38^, ^39^, ^40^, ^41^ were eligible for meta‐analysis. The pooled RR (95% CI) in the tamoxifen users (*n* = 44 980) compared to the non‐tamoxifen users (*n* = 193 414) shows a significant increasing association between use of tamoxifen and risk of EC (RR: 2.03, 95% CI: 1.68–2.45; I2:76%) (Figure 2). Furthermore, fixed method analysis shows a significant increasing association between use of tamoxifen and risk of EC (RR: 1.62, 95% CI: 1.56–1.69; I2:76.2%).

### 3.4 Subgroup analysis and meta‐regression analysis

**FIGURE 3:**
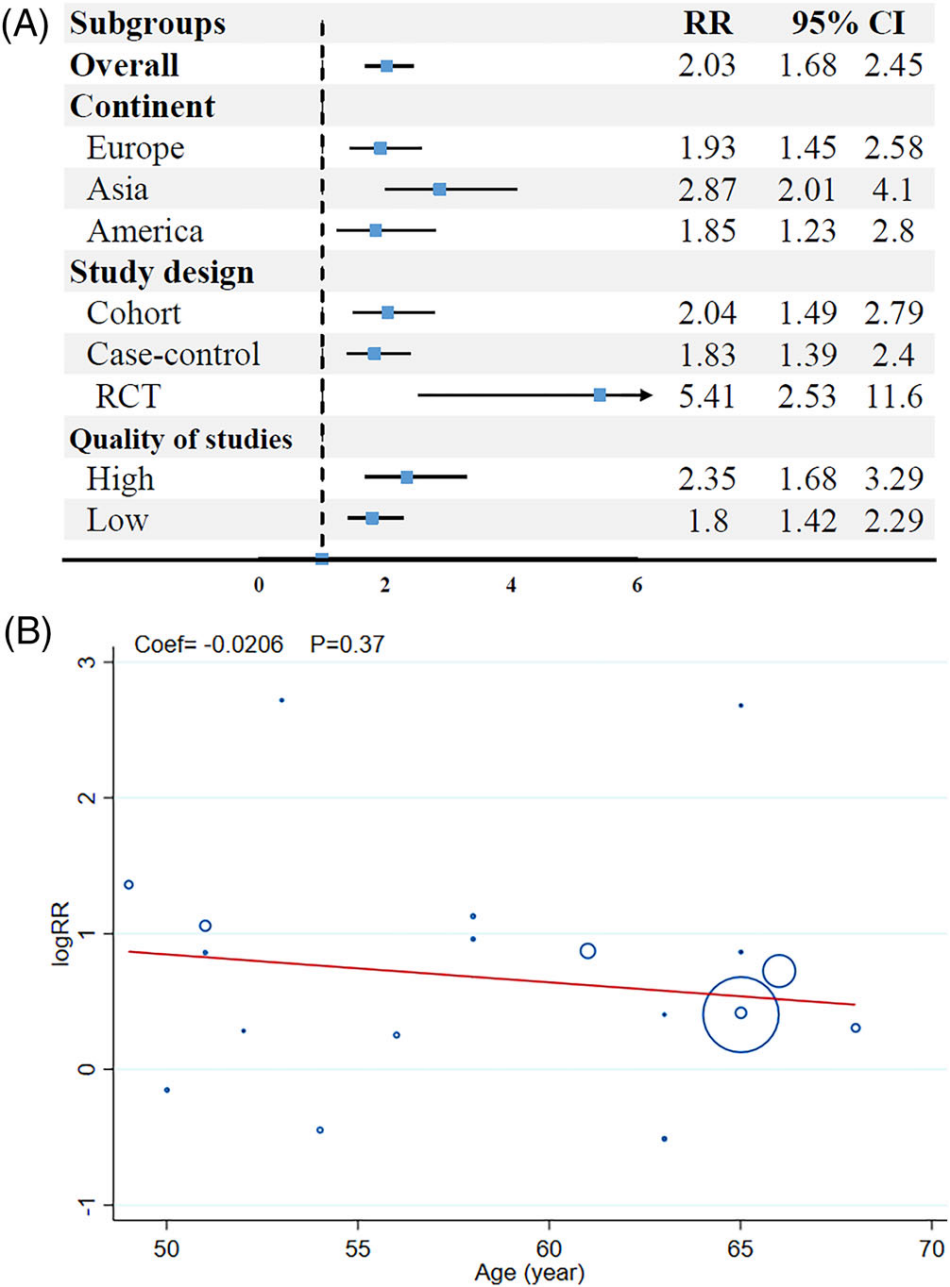
Subgroup analysis of risk ratios for endometrial cancer after tamoxifen treatment of breast cancer.

Subgroup analysis based on continent, study design and quality of studies is shown in Figure 3A. The risk of EC in tamoxifen users was 1.93 (1.45–2.53; I2:68%) in European, 2.87 (2.01–4.10; I2:20%) in Asian, and 1.85 (1.23–2.80; I2:72%) in American participants.

The most significant relation between tamoxifen use and EC in patients with BC was found in RCT studies 5.41 (2.53–11.56; I2:0%). The risk ratio of EC was 2.04 (1.49–2.79; I2:71%) in cohort studies and 1.83 (1.39–2.40; I2:66%) in case–control studies. Pooled results showed a risk of EC of 2.35 (1.68–3.29; I2:68%) in high quality studies and 1.80 (1.42–2.29; I2:75%) in low‐quality studies. According to I2 of results it is possible that the location and design of studies be as source of heterogeneity of results.

### 3.5 Meta‐regression analysis

The meta‐regression according to the age provided in the Figure 3B. The risk of EC was decrease by increase the age of participants (coef = −.0206), although this was not statistically significant (*p* = .37).

Figure S1 provides a relation between tamoxifen use and EC in patients with BC based on the year of publication. The results show no association for publication year and EC (coef = .0024, *p* = .86).

### 3.6 Dose–response analysis

**FIGURE 4:**
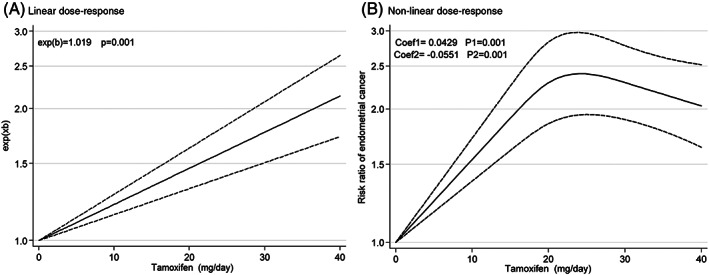
Dose–response analysis of risk ratios for endometrial cancer after tamoxifen treatment of breast cancer.

The dose–response analysis is provided in the Figure 4. The result shows significant association between tamoxifen use and EC (exe(*b*) = 1.019, *p* = .001) (Figure 4A). Furthermore, non‐linear analysis confirms a significant relation between tamoxifen and EC (Coef = .042, *p* = .001) (Figure 4B).

### 3.7 Cumulative dose–response analysis

**FIGURE 5:**
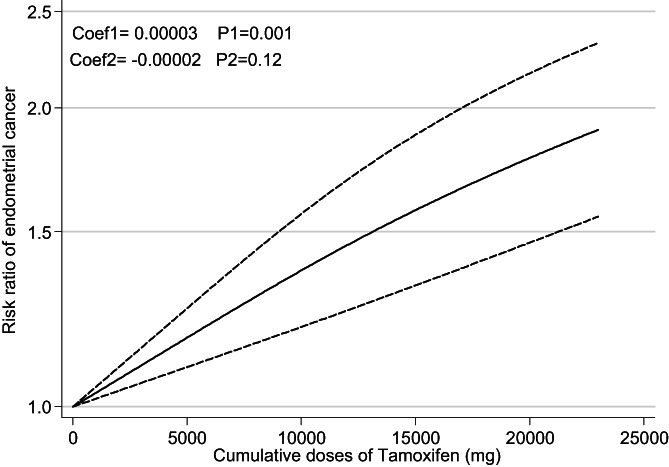
Cumulative dose–response analysis of risk ratios for endometrial cancer after tamoxifen treatment of breast cancer.

Figure 5 shows relation between cumulative dose–response tamoxifen use and risk of EC in patients with BC. Pooled results show a significant relation between cumulative dose of tamoxifen use and EC (Coef = .00005, *p* = .001).

### 3.8 Duration‐response analysis

**FIGURE 6:**
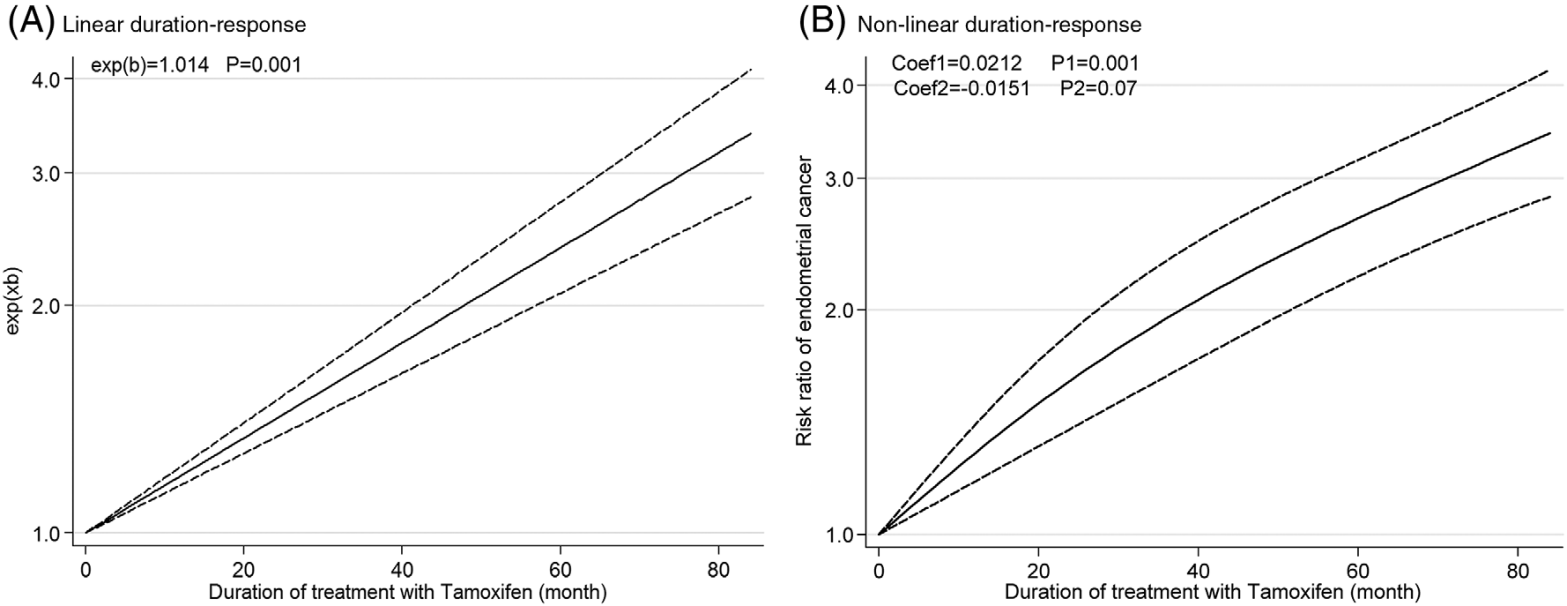
Duration‐response analysis of risk ratios for endometrial cancer after tamoxifen treatment of breast cancer.

The duration‐response analysis between tamoxifen use and EC in patients with BC is shown in Figure 6. The pooled RR from the linear and non‐linear analysis confirms a significant relation between months of tamoxifen use and EC (exe(*b*) = 1.014, *p* = .001) and (Coef = .021, *p* = .001).

### 3.9 Publication bias and single‐arm zero‐event

Sensitivity analysis of included studies is provided in Figure S2. Sensitivity analyses show that no single study had a significant impact on pooled RR and the robustness of pooled RR can be confirmed.

Although the Begg's test shows no significant (*p* = .84) publication bias among the included studies, the clear asymmetry in funnel plot (Figure S3) and significant *p* value of the Egger's regression test (*p* = .03) confirm a publication bias. Significant publication bias is evident from the results of the analysis, which show the studies that included were systematically different from those which should have been included in the analysis. The direction of studies and statistical significance of theirs results affect the publication of them, so the summary effect can be biased. Therefore, the trim‐and‐fill method was used to investigate publication bias. The trim‐and‐fill results considered 33 studies which show a risk ratio of 1.68 (1.38–2.04) for EC in patients who used tamoxifen.

Two single‐arm zero‐event studies evaluated risk of EC in related tamoxifen in BC patients.^42^, ^43^ Pooled RRs (95% CI) for tamoxifen users and non‐tamoxifen users remain significant after including them in the analysis (RR: 2.01, 95% CI: 1.66–2.42; I2:74%).

## 4 DISCUSSION

This meta‐analysis was performed to investigate the relationship between tamoxifen use and EC in patients with BC. Twenty‐six studies met inclusion criteria and were included in this analysis. Pooled analysis showed a significant increase in EC risk in patients with BC who used tamoxifen and this relation remained significant in different continent and study designs. Higher tamoxifen dose and cumulative amount of tamoxifen increase the risk of EC, independently. Furthermore, the risk of EC is dependent on duration of tamoxifen use and the longest use of it increase the risk of EC.

Tamoxifen is a SERM used for the treatment of hormone receptor positive BC as wells as for the chemoprevention in patients at higher risk of BC.^44^ Tamoxifen has both stimulating and blocking effects on estrogen receptors in the endometrium. Agonist effects of tamoxifen on the beta‐ER in the endometrium has been associated with increased risk of EC which has been associated with duration of use.^45^, ^46^ In a meta‐analysis including 21 457 patients that compared 5 years of adjuvant tamoxifen with no tamoxifen, an increased in EC risk was found to be 2.4 fold in tamoxifen users.^47^, ^48^ Longer duration of tamoxifen such as in the ATLAS and ATTom studies have found an increased risk of those using 10 years of tamoxifen compared to 5 years of tamoxifen with a cumulative risk of 1.5%–3.2% with extended therapy.^5^, ^7^ Our meta‐analysis also showed that longer use of tamoxifen increases the risk of EC as well as dose dependency with higher risk in those taking 20 mg per day.

A study reported that the risk of EC was higher in women aged 55–69 years compared to those 45 years or younger.^48^ The higher risk in postmenopausal women has been associated with a higher body mass index which is more common in older age women, which are common risk factors in general population regardless of tamoxifen use. In addition, is more common the prevalence of endometrial proliferative disorders in postmenopausal women.^9^ The age maybe has covariate role on risk of EC. In a study from the Taiwan National Health Insurance Database looking including 39 216 patients reported a higher risk of EC (HR 3.74) in women under 50 years of age.^12^ Although, currently is less common to prescribe 5 or 10 years of tamoxifen treatment for hormone receptor positive postmenopausal BC women with due to other preferred treatment strategies including aromatase inhibitors for 5 or more years, switch therapies with both aromatase inhibitors and tamoxifen, there are some premenopausal patients that are still candidates for tamoxifen treatment and also it is important to take in account that in basic resources settings tamoxifen is still the first choice of treatment in both pre and postmenopausal women.^49^, ^50^

EC is the most common gynecologic malignancy in the Western world and the most important risk factor is estrogen exposure (endogenous or exogenous) such as nulliparity, early menarche and late menopause, obesity and estrogen medication.^51^ Western women as well are those at a higher risk of tamoxifen associated EC according to our and other studies results. However, Asian women are also at risk of EC and the reason of a lower incidence might be partially explained by the median age at BC diagnosis in Asian women which is about 10 years earlier.^12^

Although the incidence of tamoxifen associated ECs is low according to our and other results and that it is diagnosed at early stage and low grade, it is important to be aware of its incidence either in premenopausal and postmenopausal women since tamoxifen is still used widely around the world specially for premenopausal patients with BC or those living in low resource settings as well as for chemoprevention in those at higher risk of BC.

This study has some limitations, including a variability of event reports based on published data which might bias results. Although, our results confirm robustness of the pooled analysis, the Egger's regression test was significant for publication bias. In this study other risk factors were not included and median age at inclusion in most of studies is above 50 years which might also bias results in the younger population.

## 5 CONCLUSIONS

This study shows there is a significant association between tamoxifen use and EC incidence in patients with BC.

## AUTHOR CONTRIBUTIONS

**Matin Ghanavati:** Conceptualization (equal); investigation (equal); writing – original draft (equal); writing – review and editing (equal). **Yasaman Khorshidi:** Data curation (equal); investigation (equal); methodology (equal); writing – review and editing (equal). **Mahdi Shadnoush:** Conceptualization (equal); project administration (equal); supervision (equal); writing – review and editing (equal). **Mohammad Esmaeil Akbari:** Conceptualization (equal); project administration (equal); resources (equal); supervision (equal); writing – review and editing (equal). **Seyed Hossein Ardehali:** Conceptualization (equal); project administration (equal); writing – review and editing (equal). **Yanin Chavarri‐Guerra:** Investigation (equal); writing – original draft (equal); writing – review and editing (equal). **Atieh Akbari:** Investigation (equal); methodology (equal); resources (equal); writing – original draft (equal); writing – review and editing (equal). **Regina Barragan‐Carrillo:** Investigation (equal); writing – original draft (equal); writing – review and editing (equal). **Manoochehr Amin Amlashi:** Data curation (equal); investigation (equal); writing – review and editing (equal). **Zeinab Javid:** Investigation (equal); methodology (equal); writing – review and editing (equal). **Jamal Rahmani:** Data curation (equal); formal analysis (lead); investigation (equal); methodology (equal); supervision (equal); visualization (equal); writing – original draft (equal); writing – review and editing (equal).

## CONFLICT OF INTEREST STATEMENT

The authors have stated explicitly that there are no conflicts of interest in connection with this article.

## ETHICS STATEMENT

Not applicable.

## Supporting information


**TABLE S1.** Electronic database search strategy
**TABLE S2.** Quality Assessment of Included Manuscripts (risk of bias assessment for RCTs).
**FIGURE S1.** Meta‐regression analysis based on year of publication of studies
**FIGURE S2.** Sensitivity analysis of included studies
**FIGURE S3.** Funnel plot publication bias

## Data Availability

The data sets used and/or analyzed during the current study are available from the corresponding author on reasonable request.
