# Supplementary material for: Tamoxifen use and risk of endometrial cancer in breast cancer patients: A systematic review and dose–response meta‐analysis
Source: Cancer Rep (Hoboken). 2023 Mar 14;6(4):e1806. doi: 10.1002/cnr2.1806 (PMC10075294; doi:10.1002/cnr2.1806)

Supplementary table 1. Electronic database search strategy

| PubMed | Scopus | WOS |
| --- | --- | --- |
| (((((((((((("Breast Neoplasms"[Mesh]) OR ("breast cancer"[Title/Abstract])) OR ("breast tumor"[Title/Abstract])) OR ("cancer of breast"[Title/Abstract])) OR ("breast carcinoma"[Title/Abstract])) OR ("breast neoplasm"[Title/Abstract])) OR ("mammary cancer"[Title/Abstract])) OR ("mammary carcinoma"[Title/Abstract])) OR ("mammary neoplasm"[Title/Abstract])) OR ("mammary tumor"[Title/Abstract])) OR ("mammary tumour"[Title/Abstract])) AND (((((((((((((((("Tamoxifen"[Mesh]) OR (tamoxifen[Title/Abstract])) OR (ICI-46,474[Title/Abstract])) OR (ICI-46474[Title/Abstract])) OR (ICI-47699[Title/Abstract])) OR (Nolvadex[Title/Abstract])) OR (Novaldex[Title/Abstract])) OR (Soltamox[Title/Abstract])) OR ("Tamoxifen Citrate"[Title/Abstract])) OR (Zitazonium[Title/Abstract])) OR (Tomaxithen[Title/Abstract])) OR (Valodex[Title/Abstract])) OR (Istubal[Title/Abstract])) OR ("N-desmethyl tamoxifen"[Title/Abstract])) OR ("4-hydroxytamoxifen"[Title/Abstract])) OR (endoxifen[Title/Abstract]))) AND ((("Endometrial Neoplasms"[Mesh]) OR ("corpus uteri"[Title/Abstract])) OR ((((((malign*[Title/Abstract]) OR (cancer*[Title/Abstract])) OR (carcinoma*[Title/Abstract])) OR (tumor*[Title/Abstract])) OR (tumour*[Title/Abstract])) AND (((endometr*[Title/Abstract]) OR ("corpus uteri"[Title/Abstract])) OR (uterine[Title/Abstract])))) | ( ( ( ( TITLE-ABS-KEY ( malign* ) OR TITLE-ABS-KEY ( cancer* ) OR TITLE-ABS-KEY ( carcinoma* ) OR TITLE-ABS-KEY ( tumor* ) OR TITLE-ABS-KEY ( tumour* ) ) ) AND ( ( TITLE-ABS-KEY ( endometr* ) OR TITLE-ABS-KEY ( uterine ) ) ) ) OR ( ( TITLE-ABS-KEY ( "Endometrial Neoplasms" ) OR TITLE-ABS-KEY ( "corpus uteri" ) ) ) ) AND ( ( TITLE-ABS-KEY ( "breast cancer" ) OR TITLE-ABS-KEY ( "breast Tumor" ) OR TITLE-ABS-KEY ( "cancer of breast" ) OR TITLE-ABS-KEY ( "breast carcinoma" ) OR TITLE-ABS-KEY ( "breast neoplasm" ) OR TITLE-ABS-KEY ( "mammary cancer" ) OR TITLE-ABS-KEY ( "mammary carcinoma" ) OR TITLE-ABS-KEY ( "mammary neoplasm" ) OR TITLE-ABS-KEY ( "mammary tumor" ) ) ) AND ( ( TITLE-ABS-KEY ( tamoxifen ) OR TITLE-ABS-KEY ( ici-46,474 ) OR TITLE-ABS-KEY ( ici-46474 ) OR TITLE-ABS-KEY ( ici-47699 ) OR TITLE-ABS-KEY ( nolvadex ) OR TITLE-ABS-KEY ( novaldex ) OR TITLE-ABS-KEY ( soltamox ) OR TITLE-ABS-KEY ( "Tamoxifen Citrate" ) OR TITLE-ABS-KEY ( "Zitazonium" ) OR TITLE-ABS-KEY ( tomaxithen ) OR TITLE-ABS-KEY ( valodex ) OR TITLE-ABS-KEY ( istubal ) OR TITLE-ABS-KEY ( "N-desmethyl tamoxifen" ) OR TITLE-ABS-KEY ( 4-hydroxytamoxifen ) OR TITLE-ABS-KEY ( endoxifen ) ) ) | (((TS=(malign* OR cancer* OR carcinoma* OR tumor* OR tumour*) AND TS=(endometr* OR uterine)) OR TS=("corpus uteri" OR "Endometrial Neoplasms")) AND TS=("breast cancer" OR "breast Tumor" OR "cancer of breast" OR "breast carcinoma" OR "breast neoplasm" OR "mammary cancer" OR "mammary carcinoma" OR "mammary neoplasm" OR "mammary tumor") AND TS=("Tamoxifen" OR "ICI-46,474" OR "ICI-46474" OR "ICI-47699" OR "Nolvadex" OR "Novaldex" OR "Soltamox" OR "Tamoxifen Citrate" OR "Zitazonium" OR Tomaxithen OR Valodex OR Istubal OR "N-desmethyl tamoxifen" OR 4-hydroxytamoxifen OR endoxifen) |

Supplementary table 2. The number of Tamoxifen use according to case and control groups.

| Study | Endometrial Cancer | | Control | | HR, OR, RR | 95% CI | | Endometrial cancer details | Race or ethnicity |
| --- | --- | --- | --- | --- | --- | --- | --- | --- | --- |
|  | All | Tamoxifen use | All | Tamoxifen use |  | LL | UL |  |  |
| Portela, S. | 45 | 23 | 49 | 15 | 14.63 | 2.43 | 87.9 | Grade 1-3.  Type 1 and 2 endometrial cancer. | Caucasian, Pakistani, Afro-Caribbean and others |
| Choi, S. | 140 | 98 | 60405 | 26936 | 2.89 | 2.01 | 4.16 | Not reported | Not reported |
| Chu, S. C. | 116 | 97 | 33774 | Not reported | 3.9 | 2.37 | 6.42 | Not reported | Not reported |
| Chiofalo, B. | 28 | 15 | 1039 | 666 | 0.64 | 0.3 | 1.37 | Not reported | Not reported |
| Guerrieri-Gonzaga, A. | 5 | 3 | 878 | Not reported | 1.33 | 0.22 | 8.04 | Not reported | Not reported |
| Chlebowski, R. T. | 107 | 89 | 7867 | 5066 | 1.36 | 0.84 | 2.22 | Not reported | Non-hispanic white, Hispanic, Black, Asian-pasific islander, others |
| Lavie, O. | 14 | 11 | 1482 | 864 | 2.62 | 0.72 | 9.44 | Uterine malignancies | Jews and Non-Jews |
| Yamazawa, K. | 6 | Not reported | 668 | Not reported | 7.92 | 0.69 | 90.89 | Not reported | Not reported |
| Swerdlow, A. J. | 813 | 665 | 1067 | 730 | 2.4 | 1.8 | 3 | Not reported | Not reported |
| Curtis, R. E. | 888 | 306 | 105753 | Not reported | 2.07 | 1.85 | 2.32 | All uterine corpus cancers | White, Black, Others |
| Pukkala, E. | 140 | 59 | 357 | 88 | 2.9 | 1.8 | 4.7 | Not reported | Not reported |
| Vrscaj, M. U. | 13 | 11 | 617 | 429 | 2.38 | 0.53 | 10.61 | Not reported | Not reported |
| Matsuyama | 12 | 9 | 6014 | 3488 | 2.37 | 0.64 | 8.77 | Not reported | Not reported |
| Bergman, L. | 299 | 108 | 860 | 245 | 1.5 | 1.1 | 1.21 | FIGO stage I to IV.  Grade 1 to 4.  Endometroid adenocarcinoma, Clear cell and papillary serous, MMMT and sarcomas of the endometrium. | Not reported |
| Peters-Engl, C. | 25 | 8 | 4084 | Not reported | 1.13 | 0.71 | 1.8 | FIGO stage I to IV.  Grade 1 to 3.  Endometroid adenocarcinoma, Adenosquamous carcinoma, Unknown. | Not reported |
| Bernstein, L. | 324 | 146 | 671 | 249 | 1.52 | 1.07 | 2.17 | Not reported | Not reported |
| Mignotte, H. | 135 | 91 | 487 | 191 | 3.1 | 1.1 | 8.7 | FIGO stage I to IV. Adenocarcinoma, Squamous carcinoma, Sarcoma, Mixed Mullerian tumor. | Not reported |
| Katase, K. | 13 | 4 | 812 | 275 | 0.86 | 0.27 | 2.8 | Not reported | Not reported |
| Sasco, A. J. | 43 | 29 | 177 | 106 | 1.29 | 0.59 | 2.79 | Stage II to IV | Not reported |
| Rutqvist, L. E. | 27 | 23 | 2702 | 1349 | 5.6 | 1.9 | 16.2 | Not reported | Not reported |
| Robinson, D. C. | 8 | 4 | 578 | 104 | 15.2 | 2.8 | 84.4 | Not reported | White and non-white |
| Cook, L. S. | 34 | 9 | 64 | 20 | 0.6 | 0.2 | 1.9 | Not reported | Not reported |
| Lahti, E. | 5 | 3 | 100 | 50 | 1.5 | 0.24 | 9.36 | Not reported | Not reported |
| Fisher, B. | 17 | 15 | 2826 | 1404 | 7.59 | 1.73 | 33.27 | FIGO stage I to IV.  Grade 1 to 3.  Endometrioid, Endometrioid, mixed, Papillary, Carcinosarcoma, Mucinous, Adenosquamous, Stromal sarcoma. | Not reported |
| andersson, M. | 9 | 7 | 1701 | 857 | 3.42 | 0.71 | 16.45 | Not reported | Not reported |
| Hardell, L. | 14 | 4 | 82 | 11 | 2.6 | 0.7 | 9.6 | Not reported | Not reported |

Supplementary table 3. Quality Assessment of Included Manuscripts (risk of bias assessment for RCTs).

| Cohort studies | selection | | | | | Comparability | Outcome | | | Total score |
| --- | --- | --- | --- | --- | --- | --- | --- | --- | --- | --- |
|  | Representativeness of the exposed cohort | Selection of the non-exposed cohort | Ascertainment of exposure | | Demonstration that outcome of interest was not present at start of study | Control for important factor or additional factor | Assessment of outcome | Was follow-up long enough for outcomes to occur | Adequacy of follow up of cohorts | Max 9 |
| Choi, S. (2021) | * | * | * | | * |  | * | * | * | 7 |
| Chu, S. C.  (2020) | * | * | * | | * | ** | * | * | * | 9 |
| Chiofalo, B. (2020) | * | * | * | | * |  | * | * | * | 7 |
| Guerrieri-Gonzaga, A.  (2016) | * | * | * | |  |  |  | * | * | 5 |
| Chlebowski, R. T. (2015) | * | * | * | | * | ** | * | * | * | 9 |
| Lavie, O.  (2008) | * | * | * | | * |  | * | * | * | 7 |
| Yamazawa, K. (2006) | * | * | * | | * |  | * | * | * | 7 |
| Curtis, R. E. (2004) | * | * | * | | * |  | * |  |  | 5 |
| Vrscaj, M. U. (2001) | * | * | * | | * |  | * | * | * | 7 |
| Matsuyama (2000) | * | * | * | |  |  | * | * | * | 6 |
| Peters-Engl, C. (1999) | * | * | * | | * | * | * | * |  | 7 |
| Katase, K. (1998) | * | * | * | | * |  | * | * | * | 7 |
| Robinson, D. C. (1995) | * | * | * | | * |  | * | * | * | 7 |
| Lahti, E. (1994) | * | * | * | | * | * | * |  |  | 6 |
| Case-control studies | selection | | | | | Comparability | exposure | | |  |
|  | Adequate definition of cases | Representativeness of the cases | Selection of Controls | Definition of Controls | | Control for important factor or additional factor | Ascertainment of exposure | Same method of ascertainment for cases and controls | Non-Response rate | Max 9 |
| Portela, S.  (2021) | * | * | * | * | | * | * | * | * | 8 |
| Swerdlow, A. J. (2005) | * | * | * | * | | ** | * | * | * | 9 |
| Pukkala, E. (2002) | * | * | * | * | | ** | * | * | * | 9 |
| Bergman, L. (2000) | * | * | * | * | | * | * | * |  | 7 |
| Bernstein, L. (1999) | * | * | * | * | | ** | * | * |  | 8 |
| Mignotte, H. (1998) | * | * | * | * | | ** | * | * |  | 8 |
| Sasco, A. J. (1996) | * | * | * | * | | ** | * | * |  | 8 |
| Cook, L. S. (1995) | * | * | * | * | | ** | * | * |  | 8 |
| Hardell, L. (1988) | * | * | * | * | | * | * | * |  | 7 |
| RCTs | Random sequence generation | Allocation concealment | Blinding of participants and personals | Blinding of outcome assessor | | Incomplete outcome data | Selective Reporting | Other bias | - | Max 7 |
| Rutqvist, L. E. (1995) | * | * | - | ? | | * | * | * |  | 5 |
| Fisher, B. (1994) | * | ? | * | ? | | * | * | * |  | 5 |
| Andersson, M. (1992) | * | ? | - | ? | | * | * | * |  | 4 |

Supplementary Figure 1. Meta-regression analysis based on year of publication of studies

Supplementary Figure 2. Sensitivity analysis of included studies


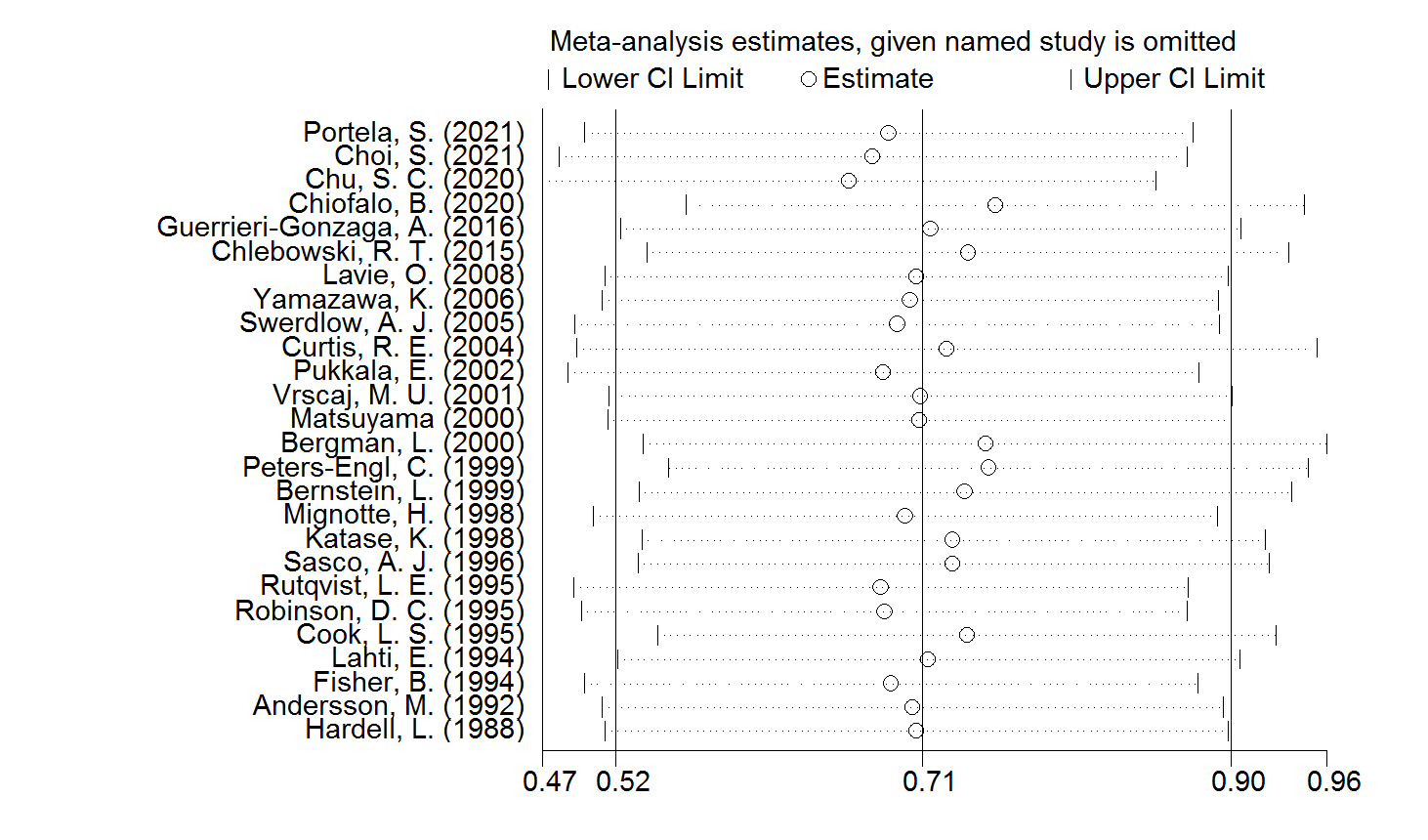


Supplementary Figure 3. Funnel plot publication bias


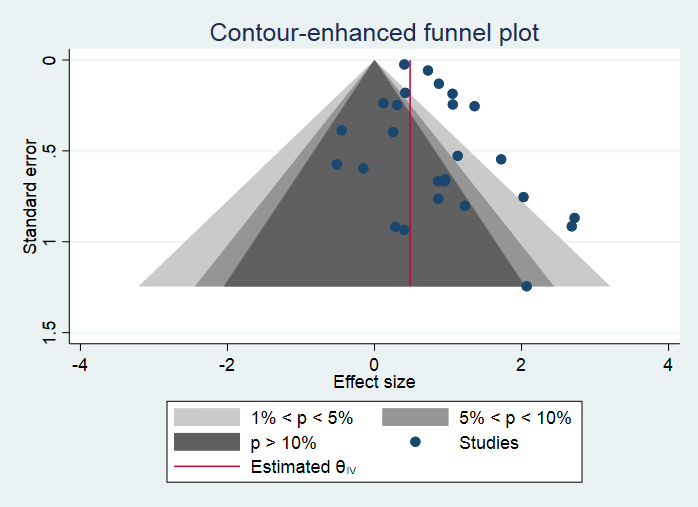

Supplement: Supplementary file 1 — TABLE S1. Electronic database search strategy TABLE S2. Quality Assessment of Included Manuscripts (risk of bias assessment for RCTs). FIGURE S1. Meta‐regression analysis based on year of publication of studies FIGURE S2. Sensitivity analysis of included studies FIGURE S3. Funnel plot publication bias [file CNR2-6-e1806-s001.docx]
